# From "best practice" to "next practice": the effectiveness of school-based health promotion in improving healthy eating and physical activity and preventing childhood obesity

**DOI:** 10.1186/1479-5868-9-27

**Published:** 2012-03-13

**Authors:** Christina Fung, Stefan Kuhle, Connie Lu, Megan Purcell, Marg Schwartz, Kate Storey, Paul J Veugelers

**Affiliations:** 1 School of Public Health, University of Alberta, 6-50 University Terrace, 8303 - 112 St, Edmonton, AB T6G 2 T4, Canada

**Keywords:** Public health, School health, Nutrition, Physical activity, Obesity, Children, Comprehensive school health, Health promotion, Program evaluation, Health policy

## Abstract

**Background:**

In 2005, we reported on the success of Comprehensive School Health (CSH) in improving diets, activity levels, and body weights. The successful program was recognized as a "best practice" and has inspired the development of the Alberta Project Promoting active Living and healthy Eating (APPLE) Schools. The project includes 10 schools, most of which are located in socioeconomically disadvantaged areas. The present study examines the effectiveness of a CSH program adopted from a "best practice" example in another setting by evaluating temporal changes in diets, activity levels and body weight.

**Methods:**

In 2008 and 2010, we surveyed grade 5 students from approximately 150 randomly selected schools from the Canadian province of Alberta and students from 10 APPLE Schools. Students completed the Harvard Youth/Adolescent Food Frequency Questionnaire, questions on physical activity, and had their height and weight measured. Multilevel regression methods were used to analyze changes in diets, activity levels, and body weight between 2008 and 2010.

**Results:**

In 2010 relative to 2008, students attending APPLE Schools were eating more fruits and vegetables, consuming fewer calories, were more physically active and were less likely obese. These changes contrasted changes observed among students elsewhere in the province.

**Conclusions:**

These findings provide evidence on the effectiveness of CSH in improving health behaviors. They show that an example of "best practice" may lead to success in another setting. Herewith the study provides the evidence that investments for broader program implementation based on "best practice" are justified.

## Background

Childhood obesity is a growing epidemic and has become a public health priority in developed countries [1,2]. Over the past few decades, prevalence rates of childhood obesity have tripled, with recent estimates indicating that 16.9% and 8.6% of children are obese in United States and Canada respectively [3-6]. Obesity negatively impacts a child's self esteem and results in diminished quality of life [7]. Moreover, children with high body mass index (BMI) often become obese adults, who are at increased risk of developing obesity-related diseases, such as type 2 diabetes, cardiovascular disease, and certain types of cancer, and place significant financial burden on healthcare systems [8-10].

Poor diets and inadequate physical activity are widely acknowledged as the main drivers of the obesity epidemic [11-13]. As childhood obesity rates continue to rise, the effects of unhealthy eating, compounded by increasingly sedentary lifestyles, emphasize the need to identify comprehensive health promotion approaches to curb the worsening trends. Recent reviews suggest the use of school-based interventions to address the childhood obesity epidemic [14,15]. Schools are the ideal setting given their ability to reach nearly all children who spend a significant proportion of their time in schools [16]. Moreover, school-based programs influence children's learning environments at a young age where healthy habits can be taught and practiced, resulting in improved health and wellness later in life [17,18].

Based on the World Health Organization's *Ottawa Charter for Health Promotion *[19], Comprehensive School Health (CSH) is an integrated school-based health promotion framework that goes beyond classroom-based health education models to a more integrated approach involving education and the whole school environment. CSH is defined as "an internationally recognized framework for supporting improvements in students' educational outcomes while addressing health in a planned, integrated and holistic way" [20]. In the United States, CSH is more commonly referred to as "Coordinated School Health"[21] while the synonymous term "Health Promoting Schools" is often used to describe the same underlying concept of creating healthier environments for children in their schools in Australia and Europe [22,23]. In addition to improvements in academic outcomes, CSH has been shown to positively influence health behaviours and health outcomes of children [17,24-26]. In 2005, we reported on the Annapolis Valley Health Promoting Schools (AVHPS) project, a successful grassroots project that achieved healthy behaviours and a reduction in the prevalence of excess bodyweight among children in Nova Scotia, Canada [27]. The successful results from the AVHPS project is now recognized as a "best practice" in Canada [28] and have inspired the development of the Alberta Project Promoting active Living and healthy Eating (APPLE) Schools in Alberta, Canada.

Despite the promising potential of CSH, only few studies have evaluated its effectiveness, and this is essential to evidence-based decision-making [22,23]. As evidence-based decision-making in public health is currently not well established, there is a strong need for rigorous studies to provide evidence on the effectiveness and sustainability of promising programs [29]. APPLE Schools, therefore, provides the opportunity to not only further evaluate the effectiveness of a CSH program for the promotion of healthy body weights among children, but to also assess the transferability of 'practice-based evidence' from a grassroots initiative to different settings within North America. The present study examines the changes in diet, physical activity, and weight status among grade 5 students in APPLE schools in comparison with students elsewhere in the province.

## Methods

### APPLE Schools: the intervention

Launched in 2008, the APPLE Schools project is a three-year intervention led by the School of Public Health at the University of Alberta. The project operates in 10 schools that were selected from five school jurisdictions in Alberta, all of which agreed to support healthy eating and active living initiatives among students. Inspired by the success of AVHPS, APPLE Schools incorporates many of the AVHPS elements and utilizes a similar CSH approach "to make the healthy choice the easy choice" [30]. However, APPLE Schools takes the AVHPS model one step further by tailoring the intervention to each of the APPLE Schools through the placement of a full-time School Health Facilitator in each school. These School Health Facilitators are responsible for implementing healthy eating and active living strategies while addressing the unique needs and barriers to health promotion in the school environment by engaging all stakeholders, including parents, staff and the community. School Health Facilitators contributed to the schools' health curriculum, both during instructional and non-instructional school time, engaged in developing cross curriculum links and taught across the curriculum. They facilitated professional development days for teachers and school staff, organized parent information nights, nutrition programs such as cooking clubs, after school physical activity programs, weekend events and celebrations, and circulated newsletters. Between 2008 and 2010, 8 of the 10 APPLE Schools implemented a nutrition policy and all 10 APPLE Schools adopted policies ensuring all their students receive a minimum of 30 minutes of physical activity per school day. Further, School Health Facilitators promoted community and parent involvement that led to community gardens, walk-to-school days, support for breakfast and lunch programs, and parent led extramural programs.

The commitment of APPLE Schools is to schools "in need". In the fall of 2007, school jurisdictions were asked to identify schools located in socioeconomically disadvantaged neighborhoods or that had otherwise challenges, included grade 5 in the grade configuration, had a principal supportive of the concept of CSH and the focus on healthy living, and schools with transient rates lower than 60%. The principals then agreed to: a) support the intervention by dedicating time directed to the project; b) commit to a three-and-a-half year involvement; c) participated in ongoing and new research; d) provide office space for the facilitator and access to infrastructure support; e) include the facilitator as part of the school staff; f) create supportive healthy living policies, and g) participate in meetings of other APPLE Schools administrators and facilitators. The recommendations by the school jurisdictions for 7 urban and 3 rural APPLE Schools with an average school size of 350 students were accommodated.

To examine the effectiveness of APPLE Schools, diet, physical activity, and health among students were measured through annual surveys using identical survey tools as the Raising healthy Eating and Active Living Kids in Alberta (REAL Kids Alberta) evaluation. In 2008, 345 home surveys and parental consent forms were distributed to parents. Of the 317 (92%) students who returned completed consent forms, 306 (97%) received parental consent to participate in the study. A total of 293 students completed the survey, resulting in a student participation rate of 85%. Similarly, data was collected among 344 and 394 consenting students and their parents from the 10 APPLE Schools in 2009 and 2010 respectively. The student participation rate in 2009 and 2010 was 84%, which is considered high for school-based research.

### REAL Kids Alberta survey

The Raising healthy Eating and Active Living Kids in Alberta (REAL Kids Alberta) is a large population-based survey that collects data on health, nutrition, physical activity, lifestyle factors, and measured height and weight among grade 5 students, and data on the school and home environment among their parents and school administrators. The aim of REAL Kids Alberta is to assess the impact of the provincial government's initiatives to promote healthy weights among children and youth in Alberta in 2008 and 2010 [31]. Details regarding the measures used as part of REAL Kids Alberta, including dietary intake, physical activity, and obesity, are provided below and are available through the project's website: http://www.REALKidsalberta.ca.

The REAL Kids Alberta evaluation used a one-stage stratified random sampling design. The sampling frame includes all elementary schools in Alberta with grade 5 students with the exception of private schools (4.7% of all Albertan students), francophone schools (0.6%), on-reserve federal schools (2.0%), charter schools (1.7%) and colony schools (0.8%) [32]. Schools were stratified according to the following geographical areas: 1) metropolitan: Calgary and Edmonton, each with populations of about 1 million people; 2) city: other municipalities with more than 40,000 residents; and 3) rural-town: municipalities with less than 40,000 residents. Schools were randomly selected within each of these geographical strata to achieve a balanced number of students in each stratum. Of the 184 invited schools, 148 (80.4%) participated in the study in 2008. Envelopes containing parental consent forms and a home survey were sent home to 5,321 students. Of the 3,704 (70%) students with completed consent forms, 3,645 (98%) received parental consent to participate in the study. Trained evaluation assistants visited each school to administer the student surveys and baseline data was collected among 3,421 students, resulting in a student participation rate of 64%. These surveys were repeated among grade 5 students of the same schools in 2010. However, within the random sample, 7 schools in 2010 refused to participate or were not available for other reasons (i.e. school closures); these schools were replaced by 10 additional schools. Therefore in 2010, 5,597 home surveys were distributed to parents from 151 randomly selected schools, of which 3,687 (66%) students returned consent forms to schools. Of these students, 3,656 (99%) received parental consent to participate and 3,469 were present to complete student surveys. A total of 3,398 participating students and their parents completed surveys, resulting in a participation rate of 61% in 2010.

### Survey Tools

#### Assessment of Dietary Intake

Students completed the Harvard Youth/Adolescent Food Frequency Questionnaire (FFQ), which has been extensively validated for use in nutrition research among children and youth 33,34. Student's caloric intake and intake of fruits and vegetables were calculated based on reported intake from the FFQ and from the Canadian Nutrient Files [35]. Overall diet quality was measured using the Diet Quality Index - International (DQI) score, a composite measure of diet quality ranging from 0 to 100 with higher scores indicating better diet quality and includes aspects of diet adequacy, variety, balance and moderation [36,37].

#### Assessment of Physical Activity

Physical activity levels were measured using the Physical Activity Questionnaire for older Children (PAQ-C), which has been demonstrated to be a valid and reliable measure of general moderate to vigorous physical activity levels over a 7-day period [38,39]. The PAQ-C score ranges from 0 to 5 with higher scores indicating higher levels of physical activity.

#### Assessment of Obesity

Student standing height was measured to the nearest 0.1 centimeter after students had removed their shoes and body weight was measured to the nearest 0.1 kilogram on calibrated digital scales. Body mass index (BMI) was calculated as weight divided by height^2 ^(kg/m^2^). Obesity was defined using the International Obesity Task Force (IOTF) BMI cut-off points that are adjusted to age and sex specific categories for children and youth [40].

#### Socioeconomic factors

Information on household income (< $50,000; $50,001 - $100,000; and > $100,000) and parental education attainment levels (secondary or less, college, university or above) were determined from household questionnaires completed by parents.

### Statistical analysis

All statistical analyses pertaining to the Alberta population were weighted to account for the design effect and represent provincial estimates of the grade 5 student population in Alberta. Differences between baseline and two-year post-intervention characteristics were assessed using the Chi-square test, Rao-Scott Chi-square or t-test where appropriate. The Rao-Scott Chi-square test was applied to examine differences in weighted estimates by adjusting for the design effect [41,42].

As observations of students are nested within those of their schools, multilevel regression methods were used to examine the effect of CSH. Odds ratios (OR) and 95% confidence intervals (CI) were calculated from multilevel logistic regression models examining the independent association of obesity with the CSH intervention. Regression coefficients (β) and 95% CI were obtained from multilevel linear regression models with fruits and vegetables consumption, dietary quality, dietary energy intake, and physical activity level as outcomes. All analyses were adjusted for the confounding potential of gender, geographic residency, household income, and parental education. Analyses pertaining to dietary intake were further adjusted for energy intake; observations with reported dietary energy intakes less than 500 kcal or more than 5,000 kcal were excluded [43]. In subanalyses, we standardized the number of servings of fruit and vegetable consumption by assuming that each child consumes 2,000 kcal each day [43]. We used the interaction term (defined as the product of the year variable and the binary intervention variable Yes = APPLE Schools, No = Provincial sample) in the adjusted multilevel models to estimate the difference in regression coefficients as a measure of intervention effect: the change among students attending APPLE Schools relative to those attending other schools in Alberta. STATA version 11 (StataCorp, College Station, TX, USA) was used to perform the statistical analysis. This study, including data collection and parental informed consent forms, was approved by the Health Research Ethics Board at the University of Alberta.

## Results

**Figure 1:**
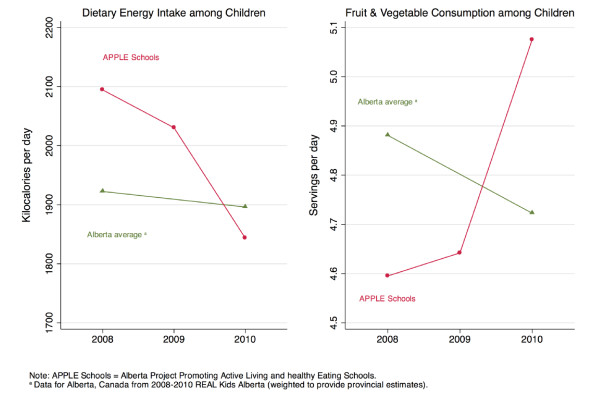
**Dietary energy intake and fruits and vegetables consumption among children by intervention exposure**.

**Figure 2:**
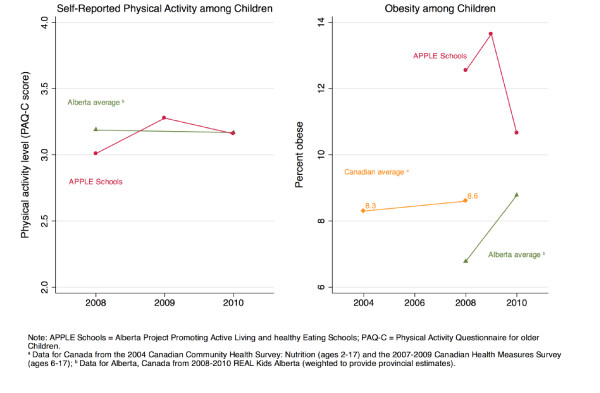
**Self-reported physical activity and prevalence of obesity among children by intervention exposure**.

Characteristics of the grade 5 students at baseline in 2008 and two-years post-intervention are shown in Table 1. With respect to gender, parental education, household income and place of residency, grade 5 students attending APPLE Schools in 2008 did not statistically differ from grade 5 students attending APPLE Schools in 2010. In 2010, relative to 2008, students attending APPLE Schools had higher intakes of fruits and vegetables, had lower caloric intakes, were more active, and were less likely to be obese (Table 1 and Figures 1 and 2). Temporal changes in provincial estimates of fruit and vegetable consumption, and caloric intake between 2008 and 2010 were less pronounced. Physical activity levels in the province increased between 2008 and 2010 but not with the same magnitude as APPLE Schools. Furthermore, in contrast to the 1.8% decline in the prevalence rates of obesity among APPLE Schools, the provincial obesity rates increased by 1.9% between 2008 and 2010 (Table 1).

**Table 1:** Characteristics of grade 5 students attending APPLE Schools and other schools in Alberta in 2008 and 2010

	APPLE Schools			Provincial^a^		
	
Independent Variable	2008	2010	*P*^b^	2008	2010	*P*^b^
*Gender*			0.10			0.42
Girls	50.7	56.8		51.5	50.5	
Boys	49.3	43.2		48.5	49.5	
*Parental Education*			0.14			0.23
Secondary or less	30.5	24.1		27.2	25.3	
College	41.1	42.8		39.7	39.4	
University or above	28.5	33.2		33.1	35.3	
*Household Income*			0.62			0.41
Less than $50,000	34.5	31.0		24.3	24.4	
$50,001 - $100,000	37.4	41.6		39.8	37.9	
>$100,000	28.1	27.4		35.9	37.7	
*Geographic Residency*			0.53			0.91
Metropolitan	65.1	62.9		46.8	46.6	
City	0.0	0.0		15.2	14.9	
Rural-town	34.9	37.1		38.0	38.5	
*Mean servings of fruits & vegetables per day*	4.60	5.08	0.02	4.88	4.73	0.09
*Mean dietary energy intake (kcal) per day*	2094	1844	< 0.01	1924	1897	0.31
*Mean DQI score*	63.2	62.3	0.30	62.8	62.5	0.23
*Mean PAQ-C score*	3.01	3.16	< 0.01	3.19	3.17	0.41
*Obese (%)*	12.5	10.7	0.45	6.9	8.8	0.01

APPLE Schools = Alberta Project Promoting active Living and healthy Eating Schools; DQI = Diet Quality Index; PAQ-C = Physical Activity Questionnaire for older Children

^a ^Estimates weighted to be representative of the grade 5 student population

^b ^p-values derived using the Chi-square test, Rao-Scott Chi-square or t-test where appropriate

After controlling for the effect of a child's gender, household income, parental education, and location of residency, multilevel regression analysis showed that students attending APPLE Schools in 2010 had better diets compared to students attending APPLE Schools in 2008, as characterized by a statistically significant increase of 0.39 serving/day in fruits and vegetables consumption, a statistically significant decrease of 237 kcal/day in dietary energy intake, and an increase in overall diet quality. Students attending APPLE Schools in 2010 were also significantly more physically active than those in 2008. Moreover, we observed a 16% decline in the odds of being obese (adjusted OR, 0.84; 95% CI, 0.52, 1.36) among students attending the APPLE Schools two years into the intervention relative to baseline (2008). In contrast, students elsewhere in the province seemed to have exhibited opposite trends over the same two-year period. Adjusted regression analysis showed that students elsewhere in Alberta saw a decrease of 0.12 serving/day in the consumption of fruits and vegetables and a decrease in diet quality. At the provincial level, no substantial changes were observed in levels of physical activity and only a modest decline in energy intake was observed. Moreover, students attending schools elsewhere in Alberta saw a 37% increase in the odds of being obese (adjusted OR, 1.37; 95% CI, 1.11, 1.70).

The change in APPLE Schools relative to the change in Alberta represents the intervention effect. The change in fruits and vegetables consumption of students attending APPLE Schools relative to those attending other Albertan schools was 0.55 serving/day and borderline significant (Table 2: 95% CI, -0.02, 1.13). APPLE Schools students' changes in physical activity and calorie consumption were also statistically significant relative to changes elsewhere in the province (Table 2). The odds of being obese in 2010 relative to 2008 was 39% lower (Table 2 OR, 0.61; 95% CI, 0.35, 1.06) among students from APPLE Schools compared to students elsewhere in the province, although this was only borderline significant. This is equivalent to a multivariable-adjusted 2.2% reduction in the prevalence of obesity among APPLE Schools between 2008 to 2010 as compared to a multivariable-adjusted 2.8% increase in the prevalence of obesity elsewhere in Alberta over the same two-year period.

**Table 2:** Effect of Comprehensive School Health on diet, physical activity and body weight among grade 5 students two years from baseline

	APPLE Schools^a^		Alberta Schools^b^		Intervention Effect: Change in APPLE Schools over time relative to the coinciding change in Alberta schools (95% CI)^b^	
Fruits and vegetables consumption per day (β and 95% CI)	0.39	(0.00, 0.78)	-0.12	(-0.29, 0.06)	0.55	(-0.02, 1.13)
Dietary energy intake (kcal) per day (β and 95% CI)	-236.51	(-366.22, -106.81)	-25.89	(-78.34, 26.56)	-212.11	(-315.07, -109.16)
DQI score (β and 95% CI)	0.96	(-0.28, 2.19)	-0.23	(-0.77, 0.31)	1.14	(-0.55, 2.83)
PAQ-C score (β and 95% CI)	0.13	(0.03,0.23)	0.02	(-0.01, 0.06)	0.10	(0.01, 0.20)
Obesity (Odds Ratio and 95% CI)	0.84	(0.52, 1.36)	1.37	(1.11, 1.70)	0.61	(0.35, 1.06)

APPLE Schools = Alberta Project Promoting active Living and healthy Eating Schools; DQI = Diet Quality Index; PAQ-C = Physical Activity Questionnaire for older Children

^a ^APPLE Schools were surveyed annually from 2008 to 2010; therefore analysis included additional measurements from 2009

^b ^Estimates weighted to be representative of the grade 5 Alberta student population

All estimates were adjusted for child's gender, household income, parental education, and rural residency. OR (Odds Ratio) were from multilevel logistic regression of obesity. β's were from multilevel linear regression representing the association of CSH programming with changes in consumption of fruits and vegetables, DQI score, dietary energy intake, and PAQ-C score. The reference category for all outcomes is 2008 baseline observations. All dietary outcomes were further adjusted for energy intake. The intervention effect was calculated using an interaction term between APPLE Schools (Yes/No) and year of observation (2008/2010). To facilitate interpretation of results, the odds of being obese was calculated from the adjusted multilevel models and converted into a probability by using the relation Prob = Odds/(Odds + 1). Data collected in 2009 in APPLE Schools was included in the multilevel regression analysis. However, 2009 observations were omitted from analyses comparing APPLE Schools with the provincial sample, as 2009 observations were not available for Alberta schools other than APPLE Schools

## Discussion

The present study demonstrates the effectiveness of a CSH intervention in fostering healthy behaviors in terms of improvements in healthy eating and active living. Over a two-year period, APPLE Schools changed their school environments and attending students reported increases in the consumption of fruits and vegetables along with decreases in energy intake, were more physically active, and exhibited less obesity compared to students elsewhere in the province.

Public health research is increasingly aiming to identify "best practice" and "practice based evidence" rather than to demonstrate universal evidence because the success of public health programs is greatly affected by contextual factors [44]. The AVHPS project, a successful grassroots project, is recognized as a "best practice" of CSH in Canada [28]. However, to our knowledge, no earlier studies have addressed the transferability of "best practice", or in other words, the extent to which the measured effectiveness of an applicable intervention could be achieved in another setting. To our knowledge, the present study is the first one where practice-based evidence of a CSH intervention was applied in a different setting while under rigorous evaluation. The demonstrated success of APPLE Schools in improving health behaviours and weight status indicates that the AVHPS model is replicable and transferable to other settings outside of the original schools in Nova Scotia, where it was developed as a grassroots initiative.

In light of the current obesity epidemic, there is a paucity of studies on the effectiveness of CSH programs [26]. Although few studies have thoroughly examined CSH interventions, our results are consistent with others that have reported on the benefits of CSH in terms of increased consumption of fruits and vegetables [45,46]. Similarly, our results are consistent with previous findings from the original AVHPS project on which the APPLE School program is based [27].

While we observed significant differences in diet and physical activity levels over a two-year period among students attending APPLE Schools, changes in obesity prevalence were only borderline significant. Longer follow-up and a larger number of schools are needed to establish improvements in longer term health outcomes such as body weights. Based on the encouraging results reported here, APPLE Schools is now expanding to include an additional 30 schools from Aboriginal and rural and remote communities throughout Alberta. This expansion will consider that schools vary in structure, organization, and objectives, and herewith that a standard implementation strategy for CSH is not plausible [23]. School Health Facilitators will be placed in new APPLE Schools as they were in the original 10 APPLE School to customize the CSH approach to suite the school's needs. By tailoring the CSH approaches to each of the APPLE Schools, the intervention builds upon ongoing health promoting activities and policies. Ongoing evaluation will further establish the benefits of CSH and the APPLE Schools approach.

The 10 APPLE Schools were selected by school jurisdictions and were mostly located in socioeconomically disadvantaged neighborhoods. That these schools were "in need" of health promotion was reflected in the poor diets and low levels of physical activity among students attending these schools at baseline in 2008. However, two years into the intervention, students attending APPLE Schools had improved their eating behaviours and physical activity levels such that they approximated or exceeded the provincial average. Given the substantial morbidity and diminished quality of life associated with poor diet, physical inactivity and childhood obesity, studies are needed to demonstrate the cost-effectiveness of CSH prevention programs considering that obese children have higher healthcare cost than normal weight children [47]. Such economic analyses will better guide public health decision makers in directing resources towards broader implementation of school-based interventions and may be instrumental in informing various policies across North America.

Strengths of the current study include its large representative sample, high response rate for school-based research, pre-intervention measurements, and the use of measured height and weight to assess body weight status. However, as with most population-based observational studies, the present study is subject to limitations. First of all, the 10 APPLE Schools were selected by school jurisdictions rather than randomly, which limits the generalizability of the results. Responses to questionnaires remain subjective and are prone to reporting error. Although individuals have a tendency to over-report levels of physical activity, it has been shown that self-reported measures of physical activity are correlated with objective measures among children [48]. Similarly, we acknowledge the imprecision associated with the assessment of dietary energy intake through the FFQ and therefore have standardized the number of servings of fruit and vegetable consumption based on energy intake. Despite the use of a validated FFQ for this age group, limitations of self-report apply to the assessment of dietary intake in which studies have shown that individuals are more likely to underreport energy intake [49]. Moreover, CSH aims to improve various aspects of the school environment such that they support improved dietary patterns and physical activity among students. The implementation was tailored and developed distinctively in each of the 10 APPLE Schools. Although randomized control trials provide the highest level of evidence for the evaluation of interventions, they may not be optimal for the evaluation of interventions that are tailored and develop distinctively. Furthermore, we opted for evaluation of prevalence rates that speak better to the needs of public health decision makers rather than incidence rates by following selected students over time.

## Conclusions

In conclusion, the APPLE Schools program demonstrated positive results in the improvement of dietary habits and physical activity levels among grade 5 students in Alberta. This suggests that the AVHPS "best practice" approach to CSH is transferable outside of the original schools in Nova Scotia to another setting, a "next practice". This study adds to the limited evidence-base of the effectiveness of CSH and justifies investments for its broader implementation.

## Competing interests

The authors declare that they have no competing interests.

## Authors' contributions

CF conducted the analyses and wrote the manuscript. PJV conceived and supervised the study. SK, CL, MP, KS, and MS advised on the analyses and contributed to the development of the manuscript. All authors read and approved the final manuscript.
